# The geopolitics of militarism and humanitarianism

**DOI:** 10.1177/03091325211032267

**Published:** 2021-09-01

**Authors:** Killian McCormack, Emily Gilbert

**Affiliations:** University of Toronto, Canada

**Keywords:** geopolitics, humanitarianism, militarism, political geography, security, war

## Abstract

In this article, we trace the interconnections between humanitarianism and militarism. We highlight the significance of a geographical perspective in emphasizing the spatial and multiscalar dimensions of this changing relationship, particularly in Western states. In doing so, we reveal the violent geographies produced through militarized humanitarianism and demonstrate the ways political violence can be obscured through invocations of humanitarianism. We look at five overlapping lines of enquiry: the way humanitarianism is used to modulate war; the rationalization of military intervention as humanitarian; military deployment in response to humanitarian crises; the military take-up of humanitarian-style practices; and weapons development and humanitarianism.

## I Introduction

Militarism is a seemingly ubiquitous feature of contemporary society. As Anna Stavrianakis and Jan Selby remark, ‘militarism – as the social and international relations of the preparation for, and conduct of, organized political violence – is an abiding and defining characteristic of world politics’ (Stavrianakis and Selby, 2013: 3; see also Stavrianakis and Stern, 2018). It is evident in the global increase in defence spending and in enduring and diverse militaristic cultures, which manifest in disparate ways in different parts of the world in different eras (Mabee and Vucetic, 2018). With the build-up of armies and arsenal, a military response to events becomes ever more likely. But, as Chris Rossdale (2019: 67) reminds us, militarism is not just about more militaries, but the whole ‘social system of values and practices which promote and underpin the use of military approaches to a vast range of situations’. As Jennifer Terry (2017: 3) argues, we are attached to war ‘not only in visibly apparent ways but in subtle and insidious ways as well’. Whether subtle or overt, militarism is inherently geographical: as Rech et al. (2015: 47) have argued, ‘there is a spatiality inherent and active in the processes [of militarism] which bring these phenomena into being, and in turn these phenomena operate to shape places, spaces, environments, and landscapes’. We see this, for example, in the militarization of national borders and policing, the production of detention and carceral spaces and the proliferation of foreign military deployments. Militarism is thus not just about the military, but the normalization and routinization of conflict and war within society, in ways that encroach upon political systems, get taken up in values and moral attachments and extend into what are otherwise usually considered to be civilian domains.

In this article, we examine how militarism intersects with humanitarianism, arguing that one way that militarism is normalized and routinized is through its entanglement with humanitarianism, especially in the West over the last few decades. Conventional definitions of humanitarianism suggest it is a moral discourse centred around a presumed universal humanity, rooted in a collection of practices of aid and care that are driven by a neutral desire to ‘do good’ and an apolitical compassion for the suffering of others. This definition would seem to position humanitarianism and militarism as quite separate endeavours. This understanding of humanitarianism, however, has received ample critique that illustrates the inherent contradictions of its moral narrative of universalism (e.g. Edkins, 2003; Skinner and Lester, 2012; Zehfuss, 2012). Michael Barnett (2011: 8) also shows how it is a sphere of global governance that is ‘increasingly public, hierarchical, and institutionalized’, which, as Simon Reid-Henry (2013) reminds us, is always connected to wider market and state practices. Whereas for Didier Fassin (2011), humanitarianism is fundamentally a politics of life in which some lives are prioritized over others, and some causes are advocated over others. These contradictions within humanitarianism come fully into view when we examine its nexus with militarism, as we will proceed to illustrate in this paper.

This nexus is not new, but is in fact fundamental to humanitarianism – as many scholars have now established. The origins of humanitarianism as a discourse have been traced to the early 19th century, in nascent forms of Western liberal internationalism that were geared to foreign assistance and steeped in religious zeal and colonialism (Barnett, 2011: 20; see also Slim, 2001; Asad, 2015). Neta Crawford (2002: 201–202) makes the more pointed argument that ‘colonialism wore two humanitarian faces’: aggressive and reformist, both of which entailed the use of ‘naked force’, either to destroy or to discipline. As a field of intervention, however, humanitarianism is said to have been founded in Henry Durant’s attempts in 1859 to provide relief to soldiers wounded on the battlefield of the Second Italian War of Independence; his efforts would lead to the formation of the International Committee of the Red Cross in 1864 and the Geneva Convention of the following year (Barnett, 2011: 1). War and humanitarianism are thus intimately linked, but this has perhaps been most pronounced post-WWII. A second era of humanitarianism emerged in this period (Barnett, 2011), which Mahmood Mamdani has described as the age of a ‘new humanitarian order’ (Mamdani, 2009). International development assistance flourished at this time as a response to waves of decolonization, but also represented lingering imperialism and was used in attempts to bring allies on side during the Cold War, while being grounded in racist and ‘(neo)colonial relations between the global North and South’ (Duffield, 2007; Hong, 2015: 13). From the 1980s, a third era of humanitarianism has been identified, with an explosion of humanitarian organizations and activities, both public and private. This has coincided with an increased state role, often manifested through the military which provides logistical support and armed protection, but also more private sector mobilization and intervention, resulting in more violence (Barnett, 2011: 4; Weizman, 2011).

While these scholars have provided a strong critique of the humanitarianism-militarism nexus, their focus is mostly historical (before the 1980s) or conceptual (with some notable exceptions, e.g. Duffield, 2007; Weizman, 2011). What shape have these entanglements taken on in more recent history? How have they touched down on the ground? With what effects? These are the questions that we take up in this article. Our focus in on the West, which we argue has seen this entanglement deepen, even as Western states are often (wrongly) characterized as being less militarized, even as the military takes on greater roles internationally and at home. To address these issues, we draw upon scholarship from a range of disciplines, but as we will illustrate throughout, the work of geographers has been at the fore of examining precisely these questions (e.g. Gregory, 2010; Lopez et al., 2015; McCormack and Gilbert, 2018). As we will show, geographers have been especially effective at exposing how the politics of life that underpins humanitarianism is grounded in the colonial and imperial history of liberalism (Lowe, 2015), and how colonial and imperial histories resurface in contemporary neoliberal state formations and international relations. Drawing significantly on the work of feminist geopolitics, geographers have also attended to multiscalar dynamics that extend across states and interstate power geometries, but which also touch down in intimate ways on bodies and populations (Dowler, 2012).

What we are interested in drawing out here, then, is not the novelty of the intersection of militarism and humanitarianism – which as we have noted is not new – but rather the changing spatial dynamics of their relationship, particularly in Western states. Our analysis attends to the geographic contexts and effects of intertwined militarism and humanitarianism; to the relational dynamic between space and these processes and practices; to the intersection of scales inherent in this, with practices that act on the body imbricated with larger geopolitical processes; and to the ways they are experienced unevenly across space, dictated along lines of race, gender, class, sexuality, ethnicity and nationality. This article is organized along five lines of enquiry: (1) the way humanitarianism is used to modulate war and conflict; (2) the rationalization of military intervention and war as humanitarian; (3) military deployment in response to humanitarian crises; (4) the military take-up of humanitarian-style practices; and 5) weapons development and humanitarianism. Addressing these in turn is an attempt to parse out and critique the implications of humanitarianism’s inclusion in military intervention and war, and the creeping logic of warfare that infuses international humanitarian politics. Yet, while these can be separated for the purposes of analysis, these logics and practices operate hand-in-hand, as will be clear as we proceed. As Inderpal Grewal (2017: 9) observes, ‘it is in the juxtaposition of necropolitics and geopolitics, the “interrelation between the sovereign right to kill and the sovereign right to rescue” that constitutes modes of state power at the end of the twentieth century’. It is precisely this aspect of state power that we interrogate in this article as we examine the spatial and multiscalar dimensions of the militarism-humanitarianism nexus.

## II Humanitarianism in the Modulation of War and Conflict

Humanitarian norms for warfare are perhaps as old as war itself, articulated, for example, through principles regarding protections for civilians or captured soldiers. For instance, Hugo Grotius’s *On the Law of War and Peace*, written in the early 17th century, was a foundational text in this sense. It established principles regarding protections for civilians or captured soldiers that were codified from the nineteenth century onwards, that in effect reified the differences between these two categories of people. Following World War II, legal principles governing the norms of war were established in what has become known as International Humanitarian Law (IHL). The objective of IHL is to limit the effects of war on the basis of humanitarian reason: reason that espouses a moral solidarity with humanity, but that is founded on uneven power relations in and between states and populations which is inherently unequal in its effects (Hong, 2015). As Didier Fassin (2011) argues, the laws are grounded in a fundamental hierarchy that determines who can be saved and who can save, and these relations of care are never reciprocated. Thus, not only do such activities perpetuate inequalities and uneven power relations, but they are also wrapped up in complex imperial dynamics pitched in terms of rescue or development (Seymour, 2012). This geopolitics is suppressed under the appeals to humanitarianism. This is the trick that the humanitarianism-military nexus performs: it hides the geopolitical inequities that it sustains, and the biopolitical violence that it enacts.

The tendency has been to presume that IHL creates a more humanitarian kind of war that offers protections for human rights, but this is a position that many have come to challenge (Orford, 2003). Eyal Weizman argues that it is through IHL that violence gets calculated and managed: a ‘humanitarian minimum’ is invoked – a moderation of ‘lesser evils’ (Weizman, 2011: 4). The principles of ‘proportionality’ and ‘distinction’ that are at the core of IHL and which are intended to limit ‘collateral damage’ actually provide the terms under which violence is permitted and legitimized (Gilbert, 2015a; Kinsella, 2011). As is now widely recognized, however, these limits effectively legitimize the deaths of civilians under specific conditions, in specific places (Gilbert, 2015a). The critiques of IHL are indebted to Walter Benjamin who maps out the violences enacted in and through both state law-making and the preservation of the law (Benjamin, 1986). Law is not (only) about the protection of human rights, or emancipation. As Weizman (2011: 4) argues, the moral technologies of humanitarian law ‘have become the means for exercising contemporary violence and for governing the displaced, the enemy and the unwanted’ that extend from the national to the international.

The consequences of the growing inclusion of humanitarian law in western practices of warfare, Laleh Khalili (2013: 7) suggests, is that ‘if policy makers think that war can be waged more humanely, they may choose to wage war more often’. And it is not just IHL, but as Craig Jones (2021) has shown, broader legal regimes and the courts that are used to legitimize and extend warfare, as military lawyers decide what makes military operations legal. In other cases, overlapping legal jurisdictions are used both to assert impunity of private military contractors and to ‘other’ the victims of violence so that they cannot access the law’s protections or compensation for harm (Snukal and Gilbert, 2015). As geographers have thus emphasized, the law is wielded in a form of lawfare against the enemy, while jurisdiction is drawn around both places and individuals to enforce separations of us and them and here and there.

There is a violence to this manipulation of the law, but the IHL is also inherently flawed in that it is rooted in principles of humanitarianism that appear to be premised on ideals of a *universal* value of life, but are enshrined in a ‘strict hierarchization’ as the lives of civilians are offset against presumed military effectiveness (Douzinas, 2003: 174; see also Dillon and Reid, 2009; Duffield, 2007; Pugliese, 2013). Judith Butler (2009: 31), discussing the value of lives under conditions of war, argues that in such hierarchies there are ‘lives that are not quite lives, cast as “destructible” and “ungrieveable,”’ on the basis of categories such as race, nationality, gender, class and sexuality. These emerge out of a geopolitics of enmity, rooted in colonial and capitalist relations of power and geopolitical control and conquest (Bhungalia, 2015). These are unquestionably racist, for as Ruth Wilson Gilmore has argued, ‘Racism is a practice of abstraction, a death-dealing displacement of different into hierarchies that organize relations within and between the planet’s sovereign political territories’ (Gilmore, 2002: 16).

As geographers and others have thus shown, maintaining the liberal order of international geopolitical relations is at the core of the relationship between militarism and humanitarianism and is increasingly apparent in the justification of military action along humanitarian lines. More and more, institutions for war-making are involved in the saving of others. Humanitarianism has been wielded to implement measures intended to protect the state from external threats, and thus they are ‘as much about self-making and self-improvement’ or self-preservation, as about providing welfare to others (Grewal, 2017: 60). Miriam Ticktin (2011: 5) has illustrated how easily framings of victims of crises can shift from ‘endangered to dangerous, innocent to delinquent’. Focusing on the ‘hotspots’ of the European Union’s external Mediterranean border, Polly Pallister-Wilkins (2018) extends this analysis to highlight how humanitarianism in this case acts to recast asylum seekers – people at risk – as a perceived threat and as a risk to the liberal order.

In these ways, geopolitical relations and processes are reshaped through humanitarian practices, which in turn produce new geographies of violence. The effects of humanitarian violence are heightened in certain spaces and at specific sites that are ‘securitized’, such as the border or the nebulous ‘battlespace’, This violence bears down differently on different bodies, for example, in terms of gender (Giles and Hyndman, 2004). But they also exist in and through crisis and disaster, drawing out militarized violence, as with refugee camps and carceral sites. Attending to the spatial dynamics of the humanitarianism-militarism nexus draws out these uneven landscapes of securitization that give lie to the claims of benevolence in which they are grounded. More research could be undertaken on how sites that are not seemingly ‘securitized’ also exemplify these the dynamics, as with the emergent work on conflict and hospitals by geographers Derek Gregory and Craig Jones.

## III Rationalization of Military Intervention and War in Terms of Humanitarianism

A prominent feature of the militarization of humanitarianism has been the co-optation of humanitarian discourse – such as the advancement or protection of human rights – in the justification of military intervention, bolstered by claims of a more ethical or ‘virtuous war’ (Chandler, 2006; Der Derian, 2009; Jabri, 2007; Zolo, 2001). By the 1990s, arguments for humanitarian war were being invoked in the name of human and national security. The US, in particular, was looking for new ways to engage its military, after its professed success in the Gulf War. As Madeleine Albright infamously stated ‘What’s the point of having this superb military that you’ve always been talking about if we can’t use it?’ (quoted in Schmitt, 1999). Humanitarianism became a way to ‘use’ the military, as it was folded into rationales for interventions as in Iraq in 1991 (Operation Provide Comfort by the US, or Operative Haven in UK parlance), in Somalia in 1992, and then with UN interventions in Kosovo (1998–1999) – and persisted in the interventions in Libya (2011) and Syria (2015–present). These discourses have been steeped in Orientalist narratives of a ‘clash of civilizations’, which pit the West and its self-serving narratives of progress and modernity against Muslim-majority nations.

These moral invocations were echoed in the emergence of the responsibility to protect (R2P) of the 1990s, which brought the security of populations who are deemed to be at risk within their own states – because of genocide, crimes against humanity or war crimes, for example – to the realm of the international community. Whereas previously, sovereign states, in theory at least, were responsible for the welfare of their respective populations independent of any external interference, R2P was proposed to justify military interventions in the name of human security to protect populations from the whims of their own government. This is a rhetoric of duty and/or obligation – but is also expressed in terms of a right to intervene that casts its target population as ‘passive beneficiaries’ (Mamdani, 2010: 54). Yet R2P is grounded in state relations forged through Western colonialism that were refracted into the international liberal order: there was a bifurcation between those states and citizens of the West seen to have full sovereignty and citizenship, while countries and populations elsewhere are deemed to be warranting trusteeship or wardship (Mamdani, 2010: 56). Indeed, the power to intervene has been limited to ‘legitimate’ Western states which are imbued with moral and political authority (Reid-Henry, 2015).

The spectre of a failed or failing state where sovereignty is compromised has also loomed large and has been wielded to legitimize military intervention (Asad, 2015). Echoing earlier imperial narratives of ‘other’ or ‘anachronistic space’ (McClintock, 1995), the invocation of failed or failing states can produce a temporal and spatial imaginary that renders space and, in turn, populations as backward. Geographers have attended to how this spatial framing implies a threat to domestic population – as with the R2P narratives – as well as a presumed threat to global security and the international order, rationalizing international intervention (McCormack, 2018; Mitchell, 2010). Maya Zehfuss argues that military interventions in the name of humanity often are complicit in ‘re-enacting rather than overcoming the colonial relation, as the problem rather than the solution’ (Zehfuss, 2012: 868). Indeed, the US led ‘War on Terror’, with its intervention against the self-styled ‘Axis of Evil’, extends colonial forms of pacification and intervention (Gregory, 2004a). Another way that it does so is, as Mamdani suggests, by operating outside of legal conventions. He argues that ‘humanitarian intervention does not need to abide by the law’, as evinced by the US invasion of Iraq in 2003 (Mamdani, 2009: 623). Thus, in the ‘war on terror,’ ‘action against certain forms of violence is simultaneously being moralized and legally deregulated’ (Mamdani, 2009: 626).

The US’s Operation Enduring Freedom was also couched in the rights-based discourse of freeing women from the Taliban’s rule in Afghanistan (Hunt, 2002; Puar and Rai, 2002; Thobani, 2002). Female engagement teams (FETs) were deployed, whose job it was to frame the US intervention in Afghanistan as a moral imperative. FETs operated on two moral levels: they brought female members of the armed forces to the forefront of operations, bringing a progressive framing to military engagement (McBride and Wibben, 2012: 200), and they engaged specifically with women from local communities, ostensibly granting them a greater voice. However, FETs did not only embody a tension between masculine and feminine performances of militarism and care (Dyvik, 2017): being central to the larger counterinsurgency campaign in Afghanistan, they mobilized moral sentiments and humanitarian activities in the waging of war. In doing so, they produced new spatial relations for the counterinsurgency war by explicitly seeking to extend the military’s reach and influence to domestic spaces. This again demonstrates the multiscalar character of militarized humanitarianism, with wider geopolitical processes shaped by moral discourses – for instance, the invasion of Iraq was in part justified by calls for a ‘responsibility to act’ against the human rights violations of Saddam Hussein (Foley, 2008: 3) – and with particular geographies of war in turn produced through moral framings.

Wars carried out in the name of humanity and human rights are also always waged on the human – or, at least, on some humans – all in the name of life, or the Foucauldian formulation of ‘letting live’ (Dillon and Reid, 2009). Despite the humanitarian narratives that have propelled military intervention and war, these military engagements invariably result in mass civilian casualties and deaths, and though fought in the name of humanitarianism and humanity, they are where, paradoxically, civilians are killed in order to be saved (Jabri, 2007; Zehfuss, 2012). Geographers have attended to the granular impact of these wars. For example, and as we discuss in more detail later, new combat technologies ensure that the risk of intervention is almost completely borne by civilians on the ground, rather than the soldiers who are dropping the air-strikes that perpetuate the differential valuation of life (Shaw, 2005). Military occupation – such as the siege in Gaza – also perpetuates this violence, even as the siege is presented as a ‘humanitarian alternative to war’ (Smith, 2015: 751). In fact, the violence is perpetuated precisely through a process of economic ‘de-development’ by targeting infrastructure, but also through limiting access to land and hence livelihoods, as well as food, water, education and healthcare, which in itself constitutes humanitarian violations (see also Bhungalia, 2010; Gregory, 2004b; Harker, 2009; Smith, 2015). Ironically, the outcome is a forced reliance on humanitarian aid that prevents Palestinian autonomy and makes them vulnerable to the vagaries of international geopolitics.

Humanitarianism thus is both the cause and consequence of violence that states eke out on both their own and other populations that results in the prolongation – and not the end – of suffering (Lopez et al., 2015). Military intervention inevitably creates new forms of humanitarian crises, for instance, through the destruction of physical and service infrastructure and in resulting health crises, for example, from lack of access to clean water, or disease and epidemics (Loyd, 2009). The contours of the geographies of violence wrought through the prism of militarism and humanitarianism are complex. Yet what is consistent through the multiple scales of these geographies are the colonial, uneven power relations that render some bodies and lives more vulnerable than others, and more likely to be injured or to die, while casting some states as sovereign and others as failed or failing.

## IV Military Deployment in Response to Humanitarian Crises

The UN Security Council’s affirmation in 1990 that humanitarian emergencies are to be considered ‘a threat to international peace and security’ paved the way for an increased military role in humanitarian scenarios (Chandler, 2006: 8). Ever since, militaries have been involved in responses to humanitarian crises, such as providing logistical support to environmental catastrophe, outbreaks of disease or the movement of displaced peoples. Couched in larger imperial contexts, crises such as the Haitian Earthquake of 2010 and the Ebola epidemic in 2014 prompted large-scale responses from the US military, with personnel dispatched to offer logistical support and to administer aid. The United Arab Emirates (UAE) has become the world’s largest provider of foreign aid, which it has rolled out through its logistics infrastructure that extends across the region and beyond, for example, also providing delivery aid to Haiti after the 2010 earthquake, even while it projects its military power in the region (Ziadah, 2019). As Rafeef Ziadah illustrates, the UAE’s logistics space not only allows it to extend humanitarian assistance well beyond zones of immediate conflict, it does so by drawing together military power and humanitarian aid that are now deeply embedded in market logics of supply chain efficiencies, returns on investments and privatization (Ziadah, 2019: 1687). Other Gulf states – such as Saudi Arabia and Oman – are developing their humanitarian logistics infrastructures with similar objectives.

Military response is also mobilized domestically in response to crises, often with respect to marginalized and alienated populations as with Hurricane Katrina in 2005 where the predominantly black population was targeted by state violence (Smith, 2006), or with Indigenous activism, whether the 1991 Oka crisis in Canada or the ongoing Dakota pipeline protests in the US (Estes, 2019). Yet militaries are also undertaking pre-emptive action. For example, this is evident in Operation Continuing Promise, launched in 2008 by US Southern Command, to provide basic medical care to vulnerable communities in Latin America and the Caribbean in anticipation of potential future crisis, which Joe Bryan (2015) has shown renders humanitarianism as a means of waging war without end. In this way, humanitarian practices and spaces form a part of a larger geography of war, anticipating and pre-empting future conflict by deploying militaries in the present.

Militaries are also involved in the securitization of channels of global migration. Geographers have underlined the ways that borders, and the work they do, are extended internally and externally through appeals to humanitarian care and control, which paradoxically serves to reinforce and securitize the very national and regional borders that asylum seekers are trying to cross (İşleyen, 2018; Jones, 2016; Pallister-Wilkins, 2018). Of course, the securitization and militarization of migration is not new. But the technologies of migration management – and the forms of circumvention or resistance that are mobilized – are taking new shape and form. What begins as a ‘humanitarian’ operation can quickly devolve into a more interventionist and militarized operation of capture, containment and control. For example, both NATO and the EU have launched military operations in response to the ongoing refugee crisis in the Mediterranean. Glenda Garelli and Martina Tazzioli (2018) have argued that although their stated aim is to protect migrants and disrupt smuggler’s networks, these missions act to dissuade migration flows through the central and eastern Mediterranean and disrupt the potential of certain populations to move through space. Migrants are both the subjects to be saved, and those who are prevented from travelling – a phenomenon that is not limited to Europe (Loyd et al., 2016; Mountz, 2020). Recent moves to militarize the response to refugees have been fuelled by the conflicts and war in Middle East and Africa – paradoxically, the means of war are increasingly being used to regulate the consequences of war – but discourses around militarization have also proliferated with the anticipated rise of ‘climate refugees’ in response to climate change (Gilbert, 2012; Hartmann, 2010). In all these examples, the nexus of militarized-humanitarianism is leading to more violence, rather than its mitigation.

As Fassin and Pandolfi (2010: 12) have argued, disasters and conflict are rendered equivalent behind the moral discourse of humanitarianism, prompting military intervention or militarized responses, and leading to the ‘naturalization – or depoliticization – of war’. Citizens are turned into ‘passive beneficiaries’ of the responsibility to protect, transformed from rights-bearing agents to ‘recipients of charity’ (Mamdani, 2009: 264). Weizman (2011: 52) has noted that the blurring of roles of militaries and humanitarians has led belligerents ‘to construe aid workers as enemies, an integral part of the occupying force’, rendering them more vulnerable to attack and violence (see also Foley, 2008). In turn, this has resulted in what Mark Duffield (2012) has termed a bunkerization of the aid industry, with the presence of humanitarian organizations becoming increasingly fortified in conflict zones, further tying them to security actors and rendering the space of aid intervention also a space of security. This reflects a hardening of boundaries that characterizes militarized humanitarianism, with specific spaces of security produced that reinforce divides between those who save and those who will be saved. However, as Jennifer Fluri has shown, attempts to secure the lives of international aid workers in conflict zones can also render civilian bodies more vulnerable, for example, through the disruptions arising from highly securitized compounds, to the violations of private spaces (Fluri, 2011). Despite these vulnerabilities, military engagement in humanitarian response is used to legitimate it as a force of good, which obfuscates its lethal force. As new crises unfold, from disasters caused by climate change to health emergencies such as the COVID-19 pandemic, geographers will need to continue to interrogate the militarized responses that are marshalled in the name of humanitarianism, as they take new shape and form.

## V The Military Take-Up of Humanitarian-Style Practices

The growing institutionalization of humanitarianism has been paralleled by a deep entanglement of biomedicine with practices of warfare, evident in counterinsurgency operations and Special Forces activities. Of course, this entanglement is not new. As noted above, early humanitarian interventions by Henry Durant were prompted by wanting to provide medical care to wounded soldiers. Indeed, war in the era of modern combat, dating from the early nineteenth century, ‘is often credited as the necessary condition under which physicians and scientists made great medical advancements in blood-banking procedures, surgical techniques, pain management, triage measures, and prosthetic rehabilitation’ (Terry, 2017: 14). Imperial military projects also hinged on medical intervention. As Bobby Wintermute (2011: 121) has shown, following the end of the Spanish-American War ‘the Army’s medical personnel were America’s primary contact with its new imperial subjects’ as military medicine was employed in the attempted pacification and subjugation of the native population of its newly acquired territories. Warwick Anderson (2006: 71) has argued that this overlap between military and medical humanitarian practices in the Philippines aimed ‘to occupy and organise a territory and a people, cultivating new forms of life, regenerating customs and habits’. Similarly, health and sanitation programmes were implemented and managed by the US Army Medical Corps with the coerced signing of the Haitian-American Convention, on the heels of US military occupation beginning on 28 July 1915 (Lopez, 2015). However, whatever health improvements were achieved were paltry and were accompanied by violence and political turmoil.

Jennifer Terry describes in detail how the military continues to make enormous investments in medical research, from prosthetics, to neurology, to pharmaceuticals. We see these dynamics at play in the contemporary moment as well, with respect to the provision of healthcare that accompanied the military reconstruction efforts in Afghanistan and Iraq (Terry, 2017: 47–48). This has included allegories of medical intervention (Bell, 2012), as with statements by military advisor David Kilcullen that invoked immunology, with the goal of counterinsurgency being ‘to restore the population’s immune system following an alien infection’ (Terry, 2017: 38). Such prominent medical metaphors contributed to an understanding of an emergent, metastasizing threat that in part justified the expansive geographic reach of the war on terror. Yet war, in turn, has also influenced biomedicine. As Alison Howell (2017: 140) notes: ‘medical sciences often shaped the practice of warfare, guiding the logistics of battle to improve the number of soldiers who could return to fighting after injury, or working to prevent the spread of disease’. As she explains further, both medicine and war operate through homologous logics of prevention and protection or through particular practices such as triage.

Following World War II, the rise in counterinsurgency campaigns has seen other humanitarian activities coupled with military violence in response to anticolonial insurgencies and in Cold War proxy wars. Geographers have teased out the ways that counterinsurgency operations have drawn together economic and state actors in the name humanitarianism, obfuscating the violence that ‘development’ programmes enact. For instance, Kevin Gould (2018) has shown how during the early 1960s the US and Guatemalan militaries invoked humanitarian sentiment in mobilizing infrastructure so as to extend state control in Northern Guatemala, displacing and upending local populations. The overlap between humanitarian and military practices was further institutionalized with the emergence of the United States Agency for International Development (USAID). As Jamey Essex (2014) has illustrated, USAID was introduced alongside heightened international policing, surveillance and punishment to promote Western economic development. The work of USAID complemented the military’s activities in counterinsurgency campaigns, notably in the Vietnam War; during the war new and deeper kinds of civil-military alliances were forged through their ‘clear and hold’ operations (Attewell, 2015). Whereas in southern Afghanistan, clearing operations were followed by rebuilding – a clear, hold, build mantra – that was meant to highlight the US military’s humanity, while also meeting its own strategic needs (Belcher, 2018). In Palestine/Israel, USAID was in fact the humanitarian face that legitimated ongoing violent intervention, which Lisa Bhungalia characterizes as a constant cycle of destruction, followed by development, followed by more destruction (Bhungalia, 2015: 2309).

In the 1990s, the military’s take-up of humanitarian-style roles deepened. NATO’s incursion into Kosovo was not just framed in terms of the need to provide military support for the protection of the population: the military went beyond providing logistical support to providing direct relief to the thousands of refugees (Porter, 2000). NATO forces were thus both waging war in the name of humanitarianism and taking responsibility for humanitarian response. With the (re)turn to counterinsurgency in the ‘war on terror’, the US military embraced tactics mobilized during previous imperial encounters (Greenberg, 2018) that more thoroughly embraced humanitarianism through their population-centric approach or what Kilcullen infamously referred to as ‘armed social work’ (Gregory, 2010: 165). This emphasis on war as a kind of protection of the population has infused the affirmations around the revival of counterinsurgency techniques of warfare as more ‘humane’ (Khalili, 2013: 3).

Humanitarian initiatives frequently coexist with, and sometimes buttress, traditional military force. Notably, when the US first intervened in Afghanistan in October 2001, it dropped both cluster bombs and food aid from the air; that the packages were easily confused because of their similarity in size and colour created a high risk of civilian casualties. With counterinsurgency and the shift to stability operations, civilian-military initiatives have proliferated, perhaps especially with respect to the US (Bachmann, 2018; Bell, 2012; Morrissey, 2015). Former Secretary of State Colin Powell referred to civilian humanitarian actors as ‘force multipliers’ at the beginning of the war on terror, and since then civilian agencies have increasingly been incorporated into practices of war (Bell, 2011; McCormack, 2018). In Iraq, the military reintroduced Provincial Reconstruction Teams (PRTs), used previously in Vietnam, that blurred military and humanitarian objectives (Christie, 2012). Academics were enrolled in the Human Terrain System (Gonzalez, 2009) or in more targeted projects such as the Bowman Expeditions in Oaxaca, Mexico (Wainwright, 2014), to provide deep social and cultural data for military intelligence. Such civil-military practices extend the geography of war. As with the FETs discussed earlier, they produce new relationships between civilians working with the military and civilians on-the-ground. Not only do these practices aim to produce social geographical knowledge to facilitate war making, but they extend into local and domestic spaces that are typically beyond military reach in conflict. Other civilian-military-private sector alliances rose up to face particular issues, such as the rebuilding of the Iraqi zoo, to extend humanitarianism to the animals, but also to ‘train’ Iraqis to be more humane both with respect to the treatment of animals and by absorbing more Americanized social values and lifestyles (Howell and Neal, 2012).

While these initiatives have been phased out, the inclusion of development alongside defence and diplomacy as one of the three pillars in the US’s National Security Strategy (2006) formalized the links between traditional military and humanitarian objectives (Attewell, 2015: 2258). We have not only seen more civil-military alliances, but soldiers have also taken up ‘non-kinetic’ activities more usually associated with humanitarian missions in an effort to win the ‘hearts and minds’ of the population and to prevent radicalization (Bell, 2012). This has ranged from conflict-prevention and ‘good governance’ to ‘money as a weapons system’ projects that include rebuilding and reconstruction but also providing micro-loans (Bachmann, 2010; Gilbert, 2015b). As development itself become more privatized, for example, through philanthropy (Mitchell and Sparke, 2016), these initiatives also deepen the military’s connections with the private sector.

How this humanitarianism has seeped into the greening of the military has also been of key concern to geographers, both with respect to rethinking core doctrine and with a move to make its operations more environmentally sustainable, from ‘green’ bullets to alternative energy sources (Bigger and Neimark, 2017; Gilbert, 2012). Such processes have been largely superficial, however: state militaries are some of the largest consumers of fossil fuels globally – for instance, the US military spent US$8.2 billion on fuel in 2017 (Office of the Assistant Secretary of Defense for Sustainment, 2018) – marking another dimension of the connection between geographies of war and geographies of extraction. This trend has been paralleled by green militarization, which has seen military forces enrolled in conservation activities to protect wildlife from poaching, often with lethal results (Masse et al., 2018; see also Peluso and Vandergeest, 2011). In each of these cases, militaries have taken up operations more often associated with the civilian sphere, folded them into their combat activities, and hence further blurred the lines between war and peace, which further endanger civilians in the field. This again demonstrates the wide-ranging, multiscalar geographies produced through the nexus of militarism and humanitarianism that are international in scope, grounded in state power and enacted on the lives and bodies of the population.

## VI Weapons Development and Humanitarianism

Not only has humanitarianism been invoked to justify wars, and to extend the spatial reach of warfare, but it is also intertwined with the legitimation of the means of war. This emphasizes the multiple scales at which the nexus of militarism and humanitarianism operates, moving from geopolitical discourse and processes, to the military-humanitarian practices that produce new spaces of war, to the modulation of bodily violence in warfare. Aligning with hierarchies of life embedded in IHL that delineate who can be killed, the means of killing in war are defined along a spectrum of humane and inhumane, legitimate and illegitimate, legal and illegal. As Asad (2015: 412) remarks, ‘ways of killing and dying are part of how we define the human’. He goes on to argue that some forms of killing are legitimized – for example, drone strikes – while others are seen to be *inhuman*, for example, ‘being hacked to death by a machete’. The designation of some weapons as civil and some as barbaric is historically contingent and underpinned by colonial power relations, and deeply entangled in war’s ‘becoming’, or what comes to count as war (Bousquet et al., 2020).

Nisha Shah’s (2017; 2019) work has traced the histories involved in the designation of certain rifle bullets as appropriate for use in war. Shah (2019: 216) shows how the quantification of bodily damage was used to calibrate a particular kind of bodily harm as appropriate in warfare, designating what kinds of injury ‘can be rendered militarily necessary and acceptable’. These developments did not prevent lethal weapons from being used, but instead served to govern what kinds of weapons could be used, because they are deemed to be ethical along the lines of proportionality. In contrast, arguments were made against new innovations, like the expanding Dum-Dum bullet – named after the factory in India where it was produced – because they went beyond what was deemed necessary to stop combatants (Shah, 2017: 96).

The development of ‘humane’ forms of killing has been paralleled by the development of non-lethal weapons and practices that incapacitate victims. Jasbir Puar (2017: x) has highlighted that purportedly humanitarian practices, such as those used by the Israeli Defence Forces, aim to ‘spar[e] death by shooting to maim’. Puar (ibid) identifies this ‘deliberate debilitation of a population’ as core to the ‘racializing biopolitical logic of security’, highlighting how seemingly benevolent humanitarian rationales and legal-moral frameworks can serve to justify certain forms of violence. The same is true for the development of non-lethal weapons, such as the taser gun, in the name of human interest, particularly that of the civilian (Anaïs, 2015). Though the objective of these weapons is to restrain and minimize lethality, such weapons are often deadly, as with the case of tear gas (Feigenbaum, 2017), or are used to make lethality possible in other ways (Anaïs, 2015). These rationales have also been deployed to roll out these kinds of weapons among other security agencies – for example, the use of tear gas by police and private security – for use both at home and abroad (Balko, 2013; Schrader, 2019), exemplifying the continuities across police power and war (Neocleous, 2014).

Such weapon developments that seek to identify and meet an acceptable level of violence are mirrored by wider developments to make war more ethical and humanitarian through technological innovation (De Landa, 1991; Owens, 2003). Writing about the military-industrial-media-entertainment network of the 21st century, James Der Derian notes how the US deployed a ‘virtuous war’ premised in ‘technological and ethical superiority’ and presumed to be a ‘bloodless, humanitarian, hygienic war’ – but one which he suggests is rooted in ‘a felicitous oxymoron’ that makes war palatable (Der Derian, 2009: xx–xxi). The aspiration to better forms of technology is rooted in the idea that some wars are justifiable, the conditions just have to be right.

This move to ‘humanitarian’ war through technological advancements is perhaps best encapsulated in the contemporary widespread use of drones – and it is here where geographers have made the most significant interventions in these debates (e.g. Hall Kindervater, 2016; Shaw and Akhter, 2012). As Ian Shaw has argued, the technological turn ushered in by drones has produced new geographies of war, marking an intensification of volume centric security and warfare (Shaw, 2016) while establishing absolute spatial distance between operator and target. However, the language that surrounds the justification of drones – that of targeted strikes – suggests an ethics to the practice of killing, involving a surgical precision that neutralizes only those bodies identified as threat (Gregory, 2011a; Mégret, 2013). The distance between drone operator and battlespace reinforces this ethical, seemingly humanitarian rationale behind the technology. As Elke Schwarz (2016: 71) highlights, ‘drone technology provides the medium and expertise to undertake targeted killing with a professional ethos and neutral distance’. Of course, this distance gives lie to how lives are valued in humanitarian war: the personnel who operate the drones at a distance are protected while lives on-the-ground are maimed and killed. It is important to bear in mind Ticktin’s (2011) argument that there is a fine line between bodies perceived as victims and bodies being perceived as threat in such contexts. Rather than humanitarian, Gregoire Chamayou refigures drone warfare as ‘humilitarian’: ‘it is a power that both kills and saves, wounds and heals, and it performs these double tasks in a single gesture, in an integrated manner’ (Chamayou, 2015: 139).

The development of an ethical, technological way of war has seen a move away from certain forms of killing that are not deemed humane or humanitarian by the ‘international community’. Over the last two decades, a series of arms control policies and treaties have been introduced to regulate the global circulation of small arms. Frequently, these attempts at regulation are framed in terms of human security, mirroring the language that has been used to justify the deregulation of more technologically advanced weaponry (Cooper, 2011), such as drones. Anna Stavrianakis (2019: 58) highlights that though attempts to control weapon circulation, such as the UN Arms Trade Treaty, are underpinned by motives of human security and framed by IHL, they say little about intra-Western arms trade, the rise in global military spending, and the wider entrenchment of militarism in society. Small arms control treaties do nothing to question the wider political economy of the global arms trade, and instead reproduce imperial power relations (Stavrianakis, 2011). Shampa Biswas (2014) illustrates how these relations are mirrored in the current nuclear non-proliferation regime, which similarly helps to produce and maintain a global order riven with unevenness, while at the same time depoliticizing the problem of nuclear proliferation itself.

These questions resonate with Asad’s (2015) discussion of what form of killing is defined as *humane* and who, exactly, is deemed capable of accessing certain forms of violence. We do not highlight these issues to argue that there should be a more extensive spread of access to weapons, but instead to raise questions regarding *who* has access to certain weapons and *who* gets to determine the terms under which they are deployed. Here again we see how international relations are modulated through imperial discourses that cast some states as democratic and hence responsible, while others must be prevented from gaining weapons at all costs, for example, by way of economic sanctions. Geographers have investigated some of these questions with respect to drone violence, but much more could be addressed with respect to other weapons and other technologies.

## VII Conclusion

Prominent framings of humanitarianism suggest a coherent collection of practices of aid and care that are driven by a moral, neutral desire to ‘do good’ and an apolitical compassion for the suffering of others. From this angle, benevolent practices are mobilized by actors in response to events identified as humanitarian crises and emergencies, such as environmental disasters and conflicts, and grounded in an ostensibly universal and unchanging understanding of the commonality of human life. What we have sought to show in this paper, however, is how Western forms of humanitarianism have always been entangled with militarism, and thus are inherently about violence (Slim, 2001). In the last decades, this entanglement has taken on new shape, as we have traced in this paper, with particular attention to understanding these contemporary dynamics from a spatial and multiscalar approach. Geographers have been at the fore of this work as they, and others, have contributed to better understanding the spatial particularities of militarized humanitarianism, both in its operation across various scales – from the international scale to that of the body – and in spaces of all kinds: of war and peace; of reconstruction and development; of inclusion and exclusion; of injury and protection; of danger and vulnerability; of intervention and resistance; of routinization and exception; and the spatial imaginaries that inform each of these characterizations.

The five overlapping themes we discuss in this article demonstrate the wealth of geographic work on these issues. This work converges around the role of militarism and humanitarianism in producing and justifying political violence in different sites and at different scales. There are, however, further avenues of research where a geographic perspective would be valuable. For instance, much of the work discussed here on the relationship between weapons development and humanitarianism has been from fields beyond geography, except in the realm of drone warfare. More could be done to explore other examples of weapons, or technologically innovation more broadly, and how they are deployed in times of war and peace, and on bodies here and there. Further, while examples of privatization are included throughout this article – from the rise of private military actors and domestic private security, to the privatization to development, from military-logistic supply chains to military purchases (such as fuel) and contracting (e.g. for weapons) – more could be made of these deepening intersections, as well as the impact that neoliberal forms of governance have on military and humanitarian logics and projects. Tracing these intersections could help inform yet other avenues of future research on how the militarism-humanitarianism nexus is being mobilized domestically, whether through the blurring relations between military and police power, the military’s role in various civic institutions, or as noted above, in examples of the deployment of the military internally at times of crisis, from environmental emergencies, to protests, to acts of terrorism (e.g. Estes, 2019; Howell, 2018; Neocleous, 2014). Is humanitarianism invoked in these domestic mobilizations when military power is meted out on those who are cast as enemies within, such as indigenous peoples, black people, Muslims, as well as migrants and refugees? And what are the continuities and discontinuities between these forms of violence at home and abroad? Finally, much of the work by geographers and others has focused on the Global North and its incursions into the Global South, perhaps replicating colonial narratives that posit the West as the site of origin for ideas, practices and technologies, which then get diffused to and imposed on the rest of the world. Are there other ways of framing this analysis that is less unidirectional, while still attentive to overarching inequities of power, technology and resources? And how does the militarism-humanitarianism nexus play out in the Global South, if at all, and with what effects?

While there are many areas where further research is warranted, the work discussed here has shown how invocations of humanitarianism, on both legal and moral grounds, can rationalize various forms of political violence and extend the means of war-making into new areas particularly in times of emergency and crisis. Better understanding the spatial dimensions of the militarism-humanitarianism nexus are especially important in today’s world which is besieged by civil violence, climate change, pandemics, poverty and migration crises – to name only some of the most pressing issues that we face today. For as we have illustrated, the humanitarianism-militarism nexus has been used to legitimize and inform military interventions in all these domains. It is thus in so small part responsible for war’s durability across time and space, as both ‘permanent’ (RETORT, 2005) and ‘everywhere’ (Gregory, 2011b).
